# FASTK post-transcriptional regulators – a ‘FAST-tracK’ in mitochondrial gene expression

**DOI:** 10.1042/BST20253089

**Published:** 2025-10-16

**Authors:** Justin Van Riper, Bridget J. Corsaro, Monica C. Pillon

**Affiliations:** 1 Verna and Marrs McLean Department of Biochemistry and Molecular Pharmacology, Baylor College of Medicine, Houston, TX 77030, U.S.A.; 2 Therapeutic Innovation Center (THINC), Baylor College of Medicine, Houston, TX 77030, U.S.A.; 3 Department of Structural Biology, Jacobs School of Medicine and Biomedical Sciences, University at Buffalo, SUNY, Buffalo, NY, 14203, U.S.A.

**Keywords:** FASTK, gene regulation, helix-turn-helix, mitochondria, nuclease, RAP domain, RNA

## Abstract

Fas-activated serine/threonine kinase (FASTK) proteins comprise one of the largest families of mitochondrial post-transcriptional regulators. Members are classified based on their conserved C-terminus, which shows homology with the PD-(D/E)XK superfamily of endoribonucleases. However, it is still uncertain which of these FASTK members are catalytic. The six human FASTK homologs rely on their RNA-binding activity to regulate distinct stages of mitochondrial gene expression, including early processing of nascent RNA, 3′-end messenger RNA (mRNA) maturation, ribosomal RNA (rRNA) modification, mRNA stability, and translation. Genetic and genomic studies have highlighted the crucial role of FASTK proteins in balancing the mitochondrial transcriptome and controlling oxidative phosphorylation. However, until recently, the molecular mechanisms governing their RNA metabolic activities have remained elusive. New biochemical and structural advances have provided molecular insights into the architecture and regulation of FASTK proteins. Here, we summarize the current understanding of the FASTK family’s specialized roles in gene regulation, with an emphasis on mitochondrial mRNA metabolism by the proteins FASTK, FASTK domain-containing protein 4 (FASTKD4), and FASTKD5. Additionally, we leverage recent experimental structures and artificial intelligence-based prediction models to explore the molecular organization of FASTK proteins and highlight the family’s signature C-terminus, a region essential for their RNA-binding activity.

## Introduction

The seminal discovery of the mitochondrial genome marked a new era of molecular biology, transforming our understanding of eukaryotic gene expression and clarifying the molecular basis of various mitochondrial diseases [1–3]. A hallmark of mitochondrial disorders is a deficiency in oxidative phosphorylation (OXPHOS), the primary process of energy production in animals [4]. Although most OXPHOS genes are encoded on the nuclear genome, the assembly and function of many respiratory complexes depend on the coordinated production of mitochondrial-encoded structural subunits [5,6]. In humans, the mitochondrial genome encodes for thirteen essential OXPHOS proteins (e.g. cytochrome c oxidase subunit 3 (COX3), NADH-ubiquinone oxidoreductase chain 6 (ND6), and ATP synthase F(0) complex subunit a (ATP6)) along with noncoding RNAs that facilitate their translation by the mitochondrial ribosome (mitoribosome) [5]. Nuclear-encoded RNA-binding proteins also enter the mitochondria to regulate all stages of mitochondrial gene expression, including RNA synthesis, processing, maturation, stability, translation, and decay. Fas-activated serine/threonine kinase (FASTK) proteins make up one of the largest families of human mitochondrial post-transcriptional regulators. Although the molecular details describing the role of FASTK proteins are still taking shape, the family’s RNA-binding activity is a keystone to mitochondrial OXPHOS gene control.

## Overview of the FASTK RNA-binding protein family

The original FASTK protein (also known as FAST) was identified 30 years ago in a genetic screen for Fas receptor-dependent apoptosis [7]. Its interaction with a cytosolic bait phosphoprotein, combined with its sequence similarity to well-known protein kinases, led to the hypothesis that FASTK is a novel serine/threonine protein kinase—inspiring its gene name [7]. However, FASTK is missing key molecular signatures of protein kinases, and recombinant FASTK protein fails to exhibit appreciable protein phosphorylation activity [7,8]. Instead, the FASTK protein was later associated with alternative splicing of Fas precursor mRNA (pre-mRNA) [9,10]. As a result, the FASTK protein’s role in apoptosis is generally attributed to its RNA-binding activity.

The FASTK family comprises several protein members critical for human mitochondrial function. Bacteria, archaea, and most unicellular eukaryotes lack *fastk* genes. However, most multicellular animals rely on FASTK proteins for energy production. There is a single FASTK member encoded in roundworm, two expressed in fly, and six produced in most vertebrates [11,12]. In humans, the founding *fastk* gene encodes for two protein products, a cytosolic isoform and a shorter mitochondrial isoform. To differentiate the original FASTK protein member from the broader FASTK family, we adopt the established nomenclature FASTK domain-containing protein 0 (FASTKD0) for the remainder of this review [12]. The remaining five protein homologs are known as FASTKD1–5 and predominantly localize to the mitochondria for gene control. Dysregulation of the human FASTK family is associated with autoimmune diseases and genetic mitochondrial disorders, often resulting in altered energy production along with a broad range of clinical presentations, including severe impairment to neurological, muscular, and kidney function [13–19]. Furthermore, expression of FASTK proteins is often altered in cancers, demonstrating potential cancer vulnerabilities and holding promise as novel diagnostic biomarkers [20–26]. Although the molecular basis of its disease association remains unclear, the FASTK family’s prominent RNA-binding activities suggest a potential pathogenic disruption to mitochondrial RNA processes [27–29].

## Molecular insights into FASTK post-transcriptional events

The production of recombinant FASTK family members has been historically difficult, limiting our understanding of these important post-transcriptional factors. However, new strategies in recombinant protein production have proven successful in acquiring several FASTK family members, opening the door for comprehensive biochemical and biophysical studies (Table 1) [8,30,31]. These recent advances expand on early genetic research and functional genomics analysis, offering in-depth molecular insight into how some FASTK family members function at discrete stages along the mRNA metabolic pathway. Since many FASTK family members still require further biochemical analysis, the following section provides i) a general overview of the FASTK family and ii) an in-depth summary of our current molecular understanding of FASTKD0, FASTKD4, and FASTKD5, which have been at the forefront of current biochemical research efforts.

**Table 1:** Summary of biochemical analyses of recombinant FASTK protein members.

	FASTKD0^NTD-CTD^ [8]	FASTKD4^CTD^ [30]	FASTKD5^NTD-CTD^ [31]
*Recombinant FASTK proteins*			
Organism	Human	Human	Human
Construct	76/169–549 aa	309–631 aa	28–764 aa
Expression host	*E. coli*	*E. coli*	Insect
Purification tag	His_6_-SUMO or GST	MBP-His_6_	His_6_
*In vitro FASTK experiments*			
Biophysical studies	SEC-MALS, SEC-SAXS, MST	MX	SEC
Biochemical assays	Native-PAGE, RNA protection	Native-PAGE	RNA processing
*FASTK molecular properties*			
Stoichiometry	Monomer	Monomer	n.d.
RNA activity	Binding, protection	Binding	Cleavage
RNA preference	G-rich ssRNA	Polyadenylated ND3	NCJ ssRNA
Functional residues	Arg249, Arg251	n.d.	n.d.

n.d. not determined; MX macromolecular crystallography; SEC size exclusion chromatography; MALS multi-angle light scattering; SAXS small angle X-ray scattering; MST microscale thermophoresis; PAGE polyacrylamide gel electrophoresis; ss single-stranded; MBP maltose binding protein; GST glutathione S-transferase; SUMO small ubiquitin-like modifier; NCJ noncanonical RNA junction; *E. coli Escherichia coli*

### Functional Overview of the FASTK Family

Mitochondrial gene expression begins with the transcription machinery synthesizing near-genome length polycistronic RNA from both the heavy and light strands of the double-stranded genome [32]. The heavy strand encodes most protein-coding genes, whereas the light strand harbors a single protein-coding gene. The nascent transcripts contain mRNA and rRNA, which are often interspersed with transfer RNA (tRNA) in a genomic arrangement known as the tRNA punctuation model [33]. Most RNA junctions are considered canonical and contain a tRNA. Structure-guided endoribonucleases RNase P and RNase Z process canonical junctions by recognizing the tRNA fold, thereby releasing the immature tRNA along with its flanking RNA species [34–38]. In contrast, noncanonical RNA junctions (NCJs) lack a tRNA, and the molecular mechanism governing NCJ processing remains poorly understood [31,39]. In 2025, FASTKD5 was identified as the long-sought NCJ-processing endoribonuclease, drawing renewed attention to this protein family [31]. The released transcripts subsequently undergo transcript-specific maturation, where FASTKD0 regulates formation of the ND6 mRNA untranslated region and FASTKD2 assists with 16S rRNA modification by RNA pseudouridine synthase D4 (RPUSD4) [8,40–44]. FASTKD4 and FASTKD0 act as RNA stability factors that protect mature mRNAs from degradation [27,30,39,43,45,46]. FASTKD3 promotes translation of mitochondrial mRNA transcripts [47]. Whereas FASTKD1 and FASTKD3 enhance turnover of specific mitochondrial RNA transcripts [12]. For an in-depth description of mitochondrial gene regulation, readers are directed to the following recent reviews [6,48].

### FASTKD5 and FASTKD4 in pre-mRNA Processing

**Figure 1:**
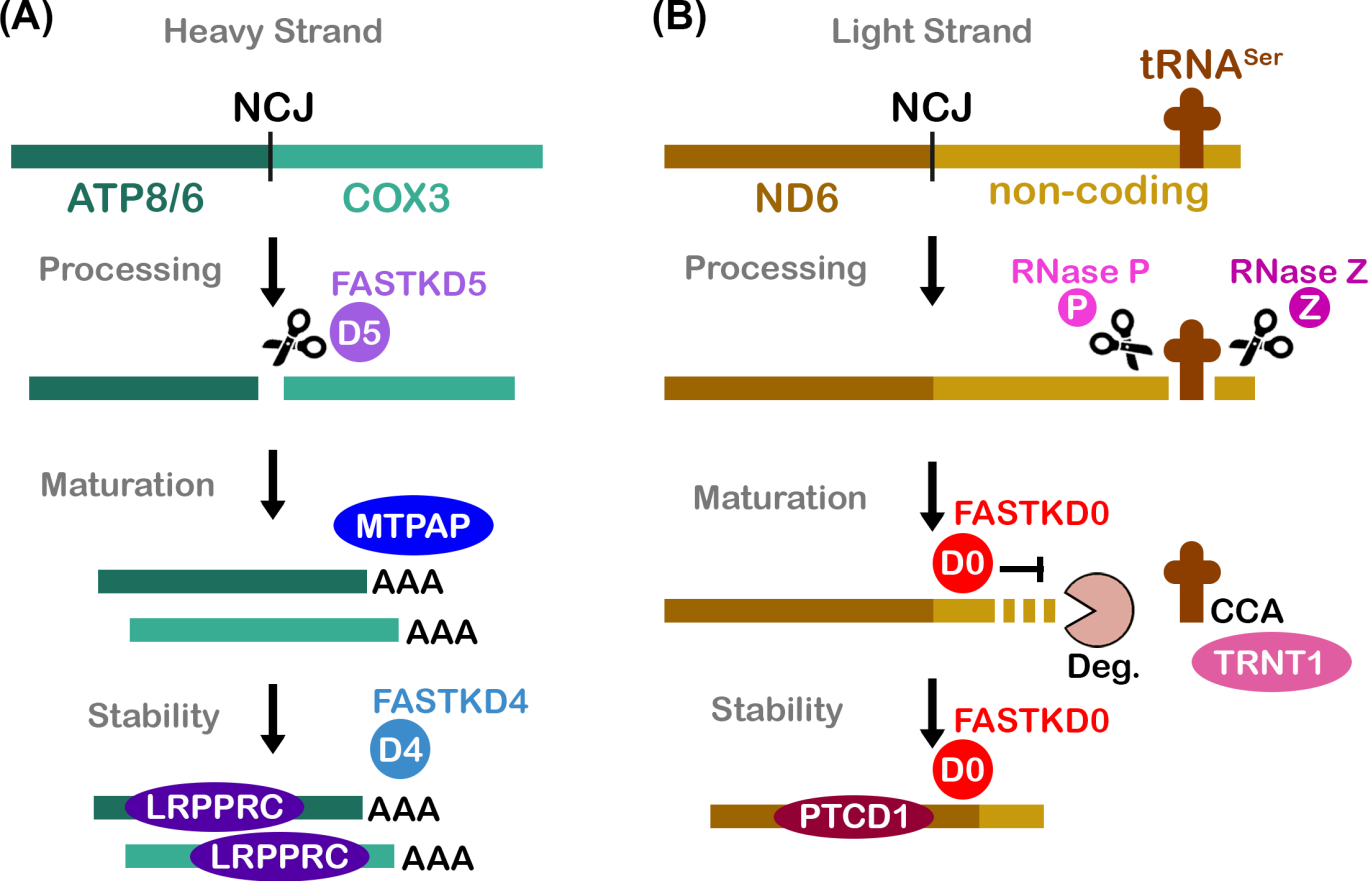
Mitochondrial gene regulation by FASTKD0, FASTKD4, and FASTKD5 RNA-binding proteins. Schematic of select mitochondrial post-transcriptional events based on the current functional understanding of biochemically characterized FASTK family members. (**A**) Polycistronic RNA derived from the heavy strand encodes for a noncanonical RNA junction (NCJ) between ATP synthase F(0) complex subunit 8/6 (ATP8/6, dark green) and cytochrome c oxidase subunit 3 (COX3, light green) mRNA transcripts. The FASTKD5 endoribonuclease (D5, purple) cleaves (scissor) at the NCJ to release the individual transcripts for subsequent 3′-end maturation resulting in the addition of a poly(A) tail by the mitochondrial poly(A) polymerase (MTPAP, royal blue) [31,49]. Additional RNA-binding proteins such as leucine-rich PPR motif-containing protein (LRPPRC, dark purple) and FASTKD4 (D4, light blue) bind to select mature transcripts to promote RNA stability [27,50]. (**B**) Nascent RNA derived from the light strand harbors the NADH-ubiquinone oxidoreductase chain 6 (ND6, brown) mRNA transcript followed by noncoding RNA (gold). Structure-guided ribonuclease proteins RNase P (P, fuchsia) and RNase Z (Z, magenta) cleave 5′ and 3′ to release a mitochondrial serine tRNA (tRNA^Ser^), respectively [34,35]. The CCA tRNA nucleotidyltransferase 1 (TRNT1, pink) subsequently modifies the 3′-end of tRNA^Ser^ whereas FASTKD0 (D0, red) binds downstream of ND6 mRNA to define a stable 3′ untranslated region during RNA trimming by the degradosome (Deg., beige) [8,51]. The RNA binding proteins pentatricopeptide repeat-containing protein 1 (PTCD1, maroon) and FASTKD0 associate with mature ND6 to promote transcript stability [52].

The FASTKD5 endoribonuclease is responsible for processing nascent polycistronic RNA at NCJs [31]. However, the molecular signatures demarcating the location of NCJs remain uncertain. Compelling biochemical evidence demonstrates for the first time that FASTKD5 exhibits endoribonuclease activity towards synthetic pre-mRNA substrates harboring unprocessed NCJs [31]. This newfound catalytic activity suggests FASTKD5 may be the elusive NCJ-processing enzyme (Figure 1A). This hypothesis is reinforced by phenotypic evidence that FASTKD5 knockout and knockdown studies result in the accumulation of unprocessed NCJ-containing transcripts along with the depletion of their mature transcripts [31,39,44,53]. Additionally, these modified cell lines display a reduction in global mitochondrial translation, consistent with a recent report that pre-mRNA processing is almost always a prerequisite for mitoribosome association [31,38,44]. FASTKD5 also co-localizes alongside nascent RNA within mitochondrial RNA granules, a region where rapid co-transcriptional RNA processing takes place [38,44]. Overall, current evidence suggests that FASTKD5 functions as a nascent polycistronic endoribonuclease responsible for releasing essential OXPHOS mRNAs—a framework supported by the embryonic lethality observed in FASTKD5 knockout mice [54]. Moving forward, it will be important to understand the molecular mechanism by which FASTKD5 recognizes NCJs.

FASTKD4 was originally hypothesized to function as an NCJ-processing enzyme; however, new findings suggest it lacks catalytic function. Like its FASTKD5 family member, FASTKD4 knockout and knockdown studies demonstrate the accumulation of unprocessed NCJ-containing transcripts and a reduction in mature transcripts [11,12,30,39,55]. Although this phenotype indicates an association with pre-mRNA processing, the loss of FASTKD4 does not strongly affect OXPHOS protein levels. Furthermore, FASTKD4 does not co-localize with nascent RNA, and recombinant FASTKD4 protein does not exhibit ribonuclease activity *in vitro* [30,44]. Consequently, FASTKD4 is more likely to indirectly facilitate NCJ-processing through an undefined mechanism.

### FASTKD5 and FASTKD0 in 3′-end mRNA Maturation

Immature mRNAs released from the heavy strand undergo 3′ polyadenylation, which often completes the stop codon and preserves transcript integrity [33,56]. While processed canonical junctions display a 3′-hydroxyl that is directly compatible for polyadenylation by the mitochondrial poly(A) polymerase (MTPAP), NCJs processed by FASTKD5 likely harbor a 3′-phosphate [31]. Intriguingly, the *Drosophila* mitochondrial Angel phosphatase binds an invertebrate FASTK homologue which is required for transcript stability, translation, and OXPHOS activity [57]. This suggests a possible role for the vertebrate ANGEL2 homologue in removing the 3′-phosphate generated by FASTKD5, thereby allowing MTPAP to append a short poly(A) tail (Figure 1A) [58]. How this multi-enzyme cascade rapidly and accurately carries out 3′-end mRNA maturation remains an open question.

The light strand encodes for a single mRNA transcript that undergoes FASTKD0-dependent 3′-end maturation. Mature ND6 mRNA lacks a poly(A) tail and instead contains a short downstream untranslated region important for stability and translation [59]. FASTKD0 binds both immature and mature ND6 mRNA transcripts, protecting its coding region and 3′-untranslated region from 3′→5′ RNA degradation by the mitochondrial degradosome (Figure 1B) [43]. Accordingly, the loss of FASTKD0 leads to a reduction in ND6 mRNA transcript levels and protein activity, whereas degradosome depletion results in the accumulation of both immature and mature ND6 mRNA [43]. Recent biochemical analysis of recombinant FASTKD0 has uncovered molecular features that likely impose, at least in part, ND6 transcript specificity. *In vitro* FASTKD0 demonstrates a binding preference for unstructured guanine-rich transcripts, potentially explaining ND6 targeting due to its high guanine content [8,60]. *In vitro* RNA protection assays further show that FASTKD0 is sufficient to shield immature ND6 transcripts from RNA degradation by the degradosome. While the field awaits RNA-bound structures of FASTKD0, basic residues within its C-terminus are important for RNA binding, and chimeric experiments swapping the C-terminus of FASTKD0 with related FASTK family members demonstrate its role in instilling transcript selectivity [8,12].

### FASTKD4 and FASTKD0 in mRNA Stability

The degradosome drives mitochondrial gene regulation through a markedly rapid but poorly understood mechanism of mRNA decay [38]. Mitochondrial leucine-rich PPR motif-containing protein (LRPPRC) acts as a general stability factor, and pentatricopeptide repeat-containing protein 1 (PTCD1) as a specialized stability factor, where they primarily bind mature transcripts to regulate differential mRNA turnover by the degradosome [38,52]. The FASTKD4 poly(A) tail mRNA-binding protein and the FASTKD0 ND6 mRNA-binding protein also serve as negative regulators of the degradosome; however, their molecular mechanisms remain to be elucidated (Figure 1) [27,30,43]. Moreover, it will be important to identify other transcript-specific stability factors to explain how mitochondria achieve their differential mRNA turnover rates. Perhaps other FASTK family members regulate transcript-specific abundance. For example, FASTKD1, FASTKD2, and FASTKD3 destabilize a variety of mRNA transcripts [12,40,43,47]. Going forward, additional genetic, biochemical, and biophysical studies are necessary to elucidate the molecular mechanisms underlying these phenotypic effects.

## Structural organization of human FASTK proteins

Alongside important advancements in our understanding of FASTK function are new insights into their molecular architecture. The first crystal structure of a FASTK member—FASTKD4—reveals the structural organization of its conserved C-terminus [30]. The solution structure of FASTKD0 also displays a similar C-terminal arrangement, suggesting the FASTK family shares a signature C-terminal fold [8]. While the field awaits additional experimental structures of FASTK RNA-binding proteins, artificial intelligence (AI) structure predictions assist with generating testable hypotheses related to the family’s architecture and regulation [61,62]. The utility of AI-based models is exemplified by their remarkable agreement with the recent crystal structure of FASTKD4 (root mean square deviation [r.m.s.d.] of 0.609 Å) [61]. Below, we leverage experimental and AI-powered prediction models of the human FASTK family to describe their overall architecture and ascribe potential function to discrete structural elements.

### Domain Organization of FASTK Proteins

**Figure 2:**
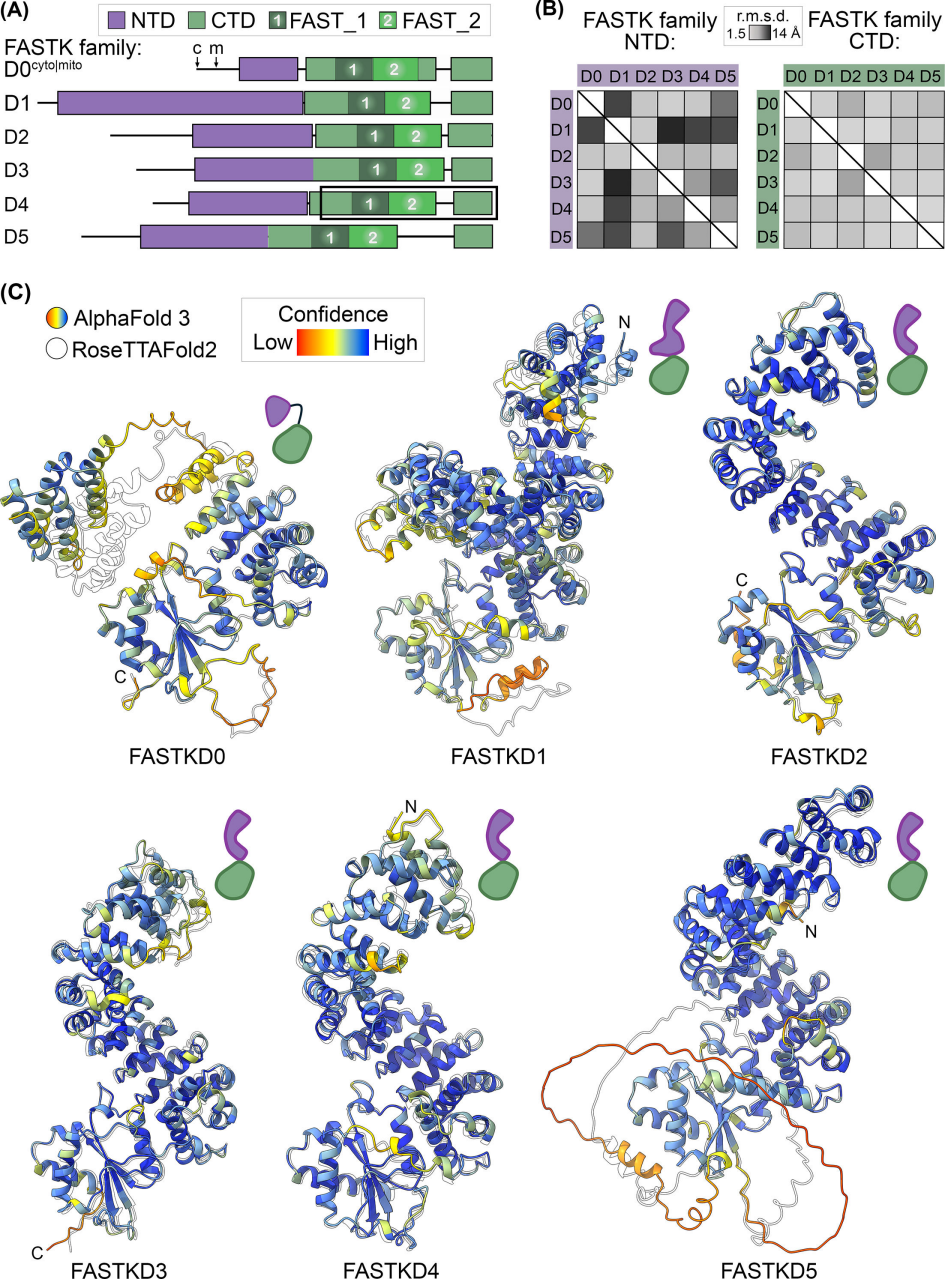
Superhelical architecture of FASTK family members. (**A**) Putative domain architecture of human FASTK family members. A HEAT-like N-terminal domain (NTD, purple) and a RAP-containing C-terminal domain (CTD, green). The domain boundaries are based on the FASTKD0 member, where a flexible linker connects discrete NTD and CTD structural folds. FAST_1 (1, dark green box) and FAST_2 (2, light green box) motifs are defined as described previously [63]. For FASTKD0, c and m demarcate the translation start site for the cytosolic and mitochondrial isoforms, respectively [43]. A black box represents the construct of the FASTKD4 crystal structure [30]. (**B**) Heat map plots summarize the r.m.s.d. between predicted FASTK NTD and CTD folds generated by AlphaFold3 [61]. (**C**) Overlay of AI-based FASTK family structure predictions generated by AlphaFold3 (color) and RoseTTAFold2 (white) [61,62]. Color gradient defines the local model confidence ranging from low (red) to high (blue) protein local distance difference test (pLDDT) [64]. Cartoons demarcate the general domain architecture of each FASTK prediction model as described in panel a.

The structural architecture of the FASTK family includes three distinct regions: a labile N-terminal tail of varying length, a helical-rich N-terminal domain (NTD), and a hallmark C-terminal domain (CTD) (Figure 2A). The flexible tail contains a subcellular targeting signal directing precursor polypeptides to the mitochondrial matrix, where they are proteolytically removed [43,63]. Proteolytically processed FASTK members are the presumed functional protein forms, relying on their NTD and CTD for mitochondrial RNA metabolism. Bioinformatic tools have predicted numerous tandem helix-turn-helix (HTH) repeats within the family’s NTD, supporting their classification as helical-repeat proteins [65]. HTH repeats are prominent structural elements in mitochondria and chloroplasts, where these well-characterized motifs (e.g. pentatricopeptide repeats) assemble into superhelical (or solenoid) structures [11,66–71]. Variations in the number of HTH repeats and stacking patterns are predicted to lead to structural diversity among the FASTK family’s NTDs (Figure 2B). Stable recombinant protein of FASTKD4’s CTD was achieved using a solubility tag, revealing for the first time a high-resolution structure of the family’s characteristic C-terminus [30]. The FASTK family’s CTD harbors a core fold typical of the RNA-binding domain abundant in Apicomplexans (RAP). Like many RAP-containing proteins, the CTD comprises two discrete regions: an upstream helical region followed by an α/β sandwich [30,72]. The helical region comprises a series of canonical HTH repeats followed by divergent helical elements commonly described as the tandem FAST_1 and FAST_2 motifs. The downstream α/β sandwich resembles the PD-(D/E)XK endonuclease-like domain superfamily [12]. The striking structural homology with well-known nucleases (e.g. very short patch repair endonuclease [Vsr]) suggests some FASTK family members may be genuine ribonucleases [31].

### NTDs Resemble Molecular Scaffolds

The FASTK family’s NTD bears resemblance to superhelical scaffolds found in higher-order mitochondrial ribonucleoprotein (RNP) assemblies. With roughly seven HTH repeats in their NTDs, most FASTK members are predicted to adopt an elongated surface with distinct concave and convex protein faces (Figure 2C). In contrast, FASTKD0 features a shorter HTH array that fails to form the characteristic inner and outer surfaces, while FASTKD1 harbors an extended array that forms a complete superhelical turn (Figure 2C). Intriguingly, the topology of most FASTK NTDs is similar to HEAT repeat proteins found within dynamic mitochondrial RNPs, such as the algae ATP synthase of *Polytomella sp. Pringsheim* and the parasitic RNA editosome of *Trypanosoma brucei* [73,74]. Notably, the editosome consists of multiple RNP assemblies, enriched with superhelical HEAT proteins that are essential for nonconventional mitochondrial mRNA processing. The FASTK NTDs align well to a core protein of the multisubunit RNA editing substrate-binding complex (RESC), known as the RESC6 HEAT repeat protein. RESC6 is an important molecular scaffold, utilizing its convex and concave surfaces to facilitate numerous interactions with neighboring RESC subunits to stabilize its higher-order assembly [75]. Moreover, RESC6 uses its concave surfaces and a composite crevasse formed with an adjacent subunit to create extended nucleic acid binding interfaces that promote noncoding RNA shielding and hybridization with pre-mRNA targets [73,75,76]. The larger FASTKD1 NTD further shares homology with RESC8, another HEAT protein that, like RESC6, forms extensive RNA contacts along its concave surface and promotes multiple contacts with nearby subunits [75,77]. While the role of the FASTK family’s NTDs is still unclear, their similarity with scaffold proteins suggests an involvement in intermolecular interactions important for specialized mitochondrial function.

### CTD Signature Forms a Putative RNA-Binding Groove

**Figure 3:**
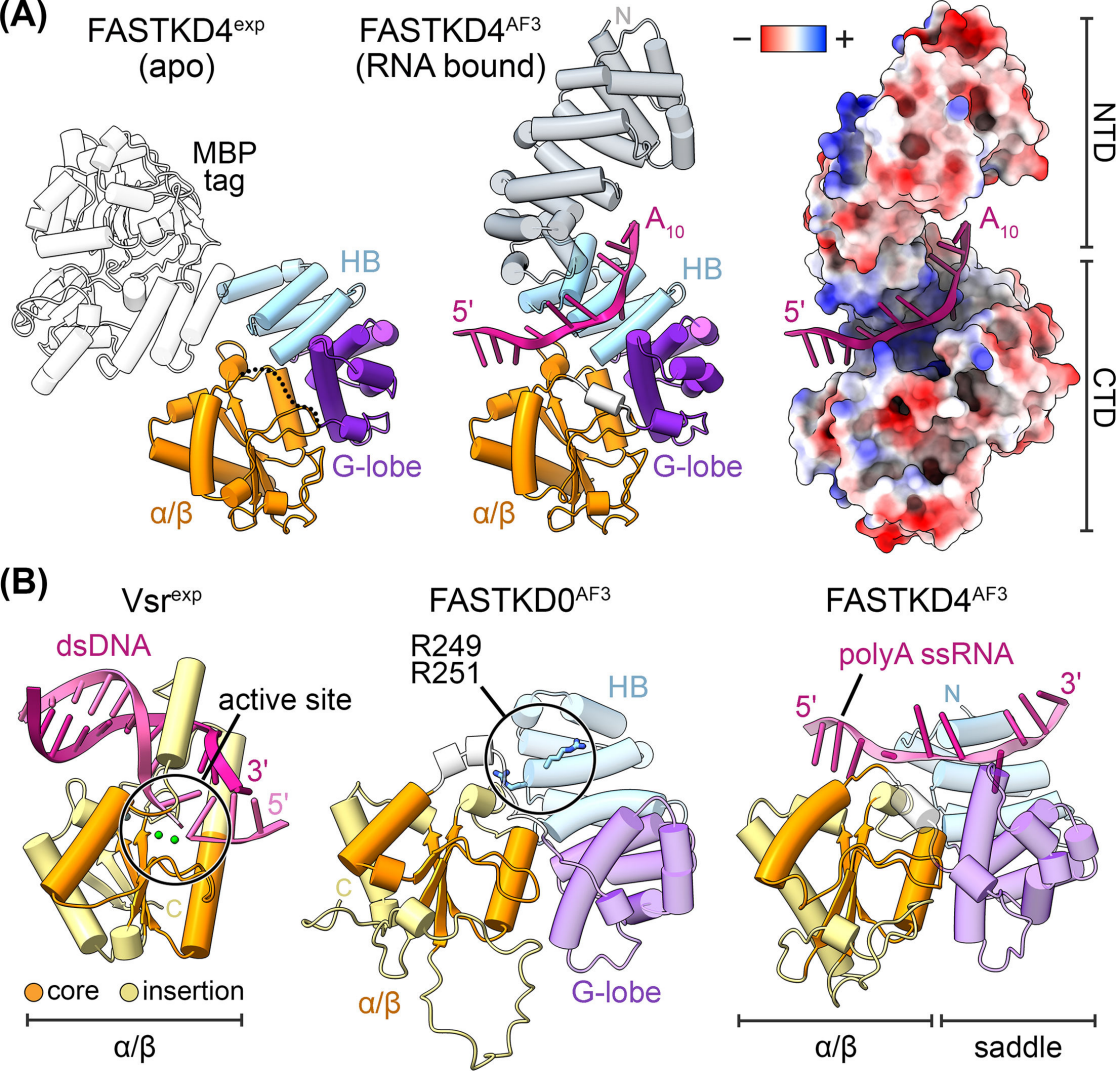
Signature C-terminal organization of FASTK proteins. (**A**) Cartoons of experimental (exp) and prediction (AF3) structures of FASTKD4 (PDB ID 9GEK (31))(58). The maltose binding protein (MBP) solubility tag is shown in white, the FASTKD4 NTD is shown in grey, and the FASTKD4 CTD is colored based on three subregions: the helical bundle (HB, blue), the groove-forming lobe (G-lobe, purple), and the RAP-like α/β sandwich (α/β, orange). The FAST_1 and FAST_2 motifs are largely contained within the HB and G-lobe elements, which together form a saddle-shaped groove large enough to accommodate unstructured RNA. The predicted 10-nucleotide poly(A) RNA substrate (**A_10_**) is shown in pink. The dotted line in the experimental structure represents a flexible unmodeled linker connecting the G-lobe and the α/β sandwich. FASTKD4 is displayed with electrostatic surface potentials, showing the putative RNA-binding surface ranging from −17 kT/e (red) to 17 kT/e (blue). (**B**) Cartoon representation of the DNA-bound Vsr crystal structure (PDB ID 1CW0 [79]) along with prediction models of the FASTKD0 and FASTKD4 CTDs [61]. The core RAP topology is shown in orange and the variable insertion elements are in yellow. The Vsr active site and FASTKD0 RNA-binding residues are circled [8,79].

The FASTK family’s CTD comprises three subregions: an upstream five-helical bundle, a groove-forming lobe, and a C-terminal RAP-like α/β sandwich. The five helical bundle shares homology with the HEAT repeat integrator 2 protein, a core structural subunit of the multiprotein Integrator complex responsible for transcription termination [78]. The helical bundle is juxtaposed to the groove-forming lobe, creating a saddle-shaped surface that is consistent in all human FASTK protein prediction models. The width and positive charge of these concave surfaces suggest they are well-suited to bind unstructured mitochondrial RNA (Figure 3A). Interestingly, FASTKD4 AI-based predictions place a short poly(A) RNA substrate along this concave surface where the helical bundle contains conserved polar residues that often score high for potential protein functionality by AlphaMissense (Figure 3A) [30,61,80]. Specifically, lysine, arginine, and serine residues line the putative RNA-binding surface, suggesting FASTKD4 may rely on strong ionic interactions to capture cognate polyadenylated RNA targets, like ND3, and counterbalance the negatively charged phosphodiester backbone [30]. Similarly, mutating equivalent basic residues within the concave surface of FASTKD0 reduces its RNA-binding activity *in vitro* [8]. Therefore, we propose that the family’s helical bundle may present important RNA-binding residues, whereas the adjacent groove-forming lobe creates a spatially restricted saddle that confers target selectivity for unstructured mitochondrial transcripts. Determining whether FASTK proteins can decipher RNA sequence within these putative RNA-binding sites to achieve transcript selectivity will be an important area of future research.

FASTK proteins exhibit a core RAP topology along with important regulatory insertion elements. Despite the superfamily’s low sequence conservation, the RAP-fold adopts a canonical αβββαβ arrangement consisting of a four-stranded β-sheet flanked by an α-helix on either side (Figure 3B). The FASTK family’s α/β sandwich forms a typical RAP fold, which along with varying insertion elements, likely instills important regulatory function to the protein members. FASTK members share homology with the RAP-containing Vsr endonuclease [79]. Vsr displays its own unique helical insertions within its RAP-fold, which clamp duplex DNA for substrate binding and cleavage (Figure 3B). The equivalent insertions necessary for Vsr DNA binding are absent from FASTK prediction models, suggesting this protein family employs an alternative mode of nucleic acid binding. Instead, FASTK proteins may rely on external structural elements, such as the putative saddle-shaped RNA-binding site. Notably, AI-generated models that thread unstructured RNA along the FASTKD4 saddle traverse the downstream RAP fold (Figure 3B). This proposed structural arrangement places the characteristic PD-(D/E)XK catalytic face in proximity to the RNA, where presumably catalytic FASTK members could cleave mitochondrial transcripts [79]. However, the extensive variability in the nuclease site arrangement across this superfamily continues to complicate the distinction between catalytic and scaffold FASTK family members. To resolve this important question, comprehensive *in vitro* studies are required to definitively identify catalytic residues and elucidate the molecular basis for their RNA metabolic activity in mitochondrial gene regulation.

## Conclusion

Substantial progress in recent years has ushered in a new era for understanding FASTK-mediated post-transcriptional regulation. Genetic approaches have had a major impact on advancing our understanding of mitochondrial gene control, whereas technical challenges associated with the recombinant production of FASTK proteins have long constrained biochemical and biophysical avenues. Recent innovations have begun to overcome these hurdles, shifting the tide to enable important FASTK molecular analyses, including much-needed structural studies. As the field continues to build its repertoire of experimental FASTK structures, predictive models—which have thus far demonstrated impressive accuracy—remain a powerful tool. Visualizing the family’s structural architecture reveals similarities to scaffold proteins, suggesting some FASTK proteins may adopt elaborate RNP assemblies, a characteristic often necessary for the selectivity of post-transcriptional regulators. AI-driven models also highlight concave surfaces within the family’s CTD, which may serve as an RNA-binding surface, paving the way for future molecular characterization of FASTK RNA recognition and metabolism.

Recent discoveries in FASTK architecture and function give rise to new questions regarding their regulation. The identity and function of protein-binding partners involved in FASTK regulation remain largely elusive. Notably, FASTKD5 has been shown to associate with the nucleotide-binding oligomerization domain (NOD) family receptor NLR family member X1 (NLRX1), altering mitochondrial RNA processing and OXPHOS activity [53,81,82]. However, the molecular basis underlying NLRX1-dependent regulation of FASTKD5 function remains unclear. Proximity labeling technology has proven fruitful in nominating protein-binding partners, including mitoribosome assembly factors (Neugrin (NGRN), RCC1-like G exchange factor-like protein (RCC1L), and RPUSD4) and the mitochondrial transcription terminator factor (MTERFD1) for FASTKD5 as well as the G-rich RNA-binding protein (GRSF1) and RNA helicases (DEAD-box helicase 28 (DDX28) and DExH-box helicase 30 (DHX30)) for FASTKD2 [83]. Furthermore, FASTKD0 co-localizes with GRSF1, suggesting a potential molecular interaction [43]. Moving forward, it will be important to identify and validate the complete repertoire of FASTK family binding partners and define their regulatory roles in discrete stages of mitochondrial RNA metabolism. Genomics and proteomics offer relatively unbiased approaches to mapping FASTK-centric interactomes. Advances in live cell preparations can label the mitochondrial proteome in its native microenvironment, producing proteomic maps that preserve important spatial organization and transient intermolecular interactions [83–85]. Popular pipelines such as cross-linking and immunoprecipitation (CLIP) with next generation sequencing can provide a glimpse into their potential RNA-binding preference and have been performed for FASTKD2 and FASTKD4 [39,86]. Building on this momentum, new adaptations to these pipelines, such as Selective RNase H-mediated interactome framing for target RNA regions (SHIFTR), aim to further describe the complex proteomes linked to specific RNA regions, potentially enabling the identification of FASTK-containing RNP complexes that function at discrete stages of the mitochondrial RNA metabolic pathway [87]. New deep learning resources such as the Encyclopedia of Domains and HydRA are expanding the known catalog of RNA-binding domains, offering an opportunity to identify FASTK RNA-binding sites [88,89]. High-throughput approaches to characterize protein–RNA interfaces, such as RNA Bind-n-Seq and RNAcompeteS, may also uncover new molecular insight into the potential role of RNA motifs in the FASTK family’s target selectivity [90–92]. By harnessing these powerful methodologies, the coming years are poised to transform our understanding of FASTK family-driven mitochondrial processes and how their dysregulation contributes to disease.

**Perspectives**

- Fas-activated serine/threonine kinase (FASTK) post-transcriptional regulators are a poorly understood family of RNA-binding proteins that play a central role in mitochondrial gene expression. Dysregulation of FASTK members is linked to autoimmune disease, mitochondrial disorders, and cancer, underscoring their role in maintaining human health.
- Advances in biochemical and biophysical studies synergize with genetic evidence to reveal discrete RNA metabolic roles of FASTK family members. Notably, FASTK domain-containing protein 5 (FASTKD5), FASTKD0, and FASTKD4 play vital roles in mitochondrial messenger RNA processing, maturation, and stability, respectively.
- The FASTK field requires an in-depth mechanistic understanding of its members’ higher-order assembly and mode of RNA target selectivity. It will be important to visualize how FASTK RNA-binding proteins distinguish their cognate targets and hand off RNA intermediates during intricate cascades of RNA processing.

## Open Access

This article has been published open access under our Subscribe to Open programme, made possible through the support of our subscribing institutions, learn more here: https://portlandpress.com/pages/open_access_options_and_prices#conditional

## Abbreviations

AI: artificial intelligence
CTD: C-terminal Domain
FASTK: Fas-activated serine/threonine kinase
FASTKD0: FASTK domain-containing protein 0
GRSF1: G-rich RNA sequence binding factor 1
HTH: helix-turn-helix
MTPAP: mitochondrial poly(A) polymerase
NCJ: noncanonical RNA junction
NTD: N-terminal domain
OXPHOS: oxidative phosphorylation
RAP: RNA-binding domain abundant in Apicomplexans
RESC: RNA editing substrate-binding complex
RNP: ribonucleoprotein
Vsr: very short patch repair endonuclease
mRNA: messenger RNA
pre-mRNA: precursor mRNA
rRNA: ribosomal RNA
tRNA: transfer RNA
